# Neurosporaxanthin Overproduction by *Fusarium fujikuroi* and Evaluation of Its Antioxidant Properties

**DOI:** 10.3390/antiox9060528

**Published:** 2020-06-16

**Authors:** Obdulia Parra-Rivero, Marcelo Paes de Barros, María del Mar Prado, José-Vicente Gil, Dámaso Hornero-Méndez, Lorenzo Zacarías, María J. Rodrigo, M. Carmen Limón, Javier Avalos

**Affiliations:** 1 Department of Genetics, Faculty of Biology, University of Seville, 41012 Seville, Spain; duly@us.es (O.P.-R.); jcymmr5@gmail.com (M.d.M.P.); carmenlimon@us.es (M.C.L.); 2 Department of Food Biotechnology, Institute of Agrochemistry and Food Technology (IATA-CSIC), 46980 Valencia, Spain; marcelo.barros@cruzeirodosul.edu.br (M.P.d.B.); J.Vicente.Gil@uv.es (J.-V.G.); lzacarias@iata.csic.es (L.Z.); mjrodrigo@iata.csic.es (M.J.R.); 3 Interdisciplinary Program in Health Sciences, Institute of Physical Activity Sciences and Sports (ICAFE), Cruzeiro do Sul University, Rua Galvão Bueno 868, São Paulo SP 01506-000, Brazil; 4 Food Technology Area, Faculty of Pharmacy, University of Valencia, Burjassot, 46100 Valencia, Spain; 5 Department of Food Phytochemistry, Instituto de la Grasa (IG-CSIC), 41013 Seville, Spain; hornero@ig.csic.es

**Keywords:** carotenoids, xanthophylls, free radicals, oxidative stress, fungi, quenching, liposomes

## Abstract

Neurosporaxanthin (NX) is a carboxylic carotenoid produced by some filamentous fungi, including species of the genera *Neurospora* and *Fusarium*. NX biosynthetic genes and their regulation have been thoroughly investigated in *Fusarium fujikuroi*, an industrial fungus used for gibberellin production. In this species, carotenoid-overproducing mutants, affected in the regulatory gene *carS*, exhibit an upregulated expression of the NX pathway. Based on former data on a stimulatory effect of nitrogen starvation on carotenoid biosynthesis, we developed culture conditions with *carS* mutants allowing the production of deep-pigmented mycelia. With this method, we obtained samples with ca. 8 mg NX/g dry mass, in turn the highest concentration for this carotenoid described so far. NX-rich extracts obtained from these samples were used in parallel with *carS*-complemented NX-poor extracts obtained under the same conditions, to check the antioxidant properties of this carotenoid in in vitro assays. NX-rich extracts exhibited higher antioxidant capacity than NX-poor extracts, either when considering their quenching activity against [O_2_(^1^Δ_g_)] in organic solvent (singlet oxygen absorption capacity (SOAC) assays) or their scavenging activity against different free radicals in aqueous solution and in liposomes. These results make NX a promising carotenoid as a possible feed or food additive, and encourage further studies on its chemical properties.

## 1. Introduction

Carotenoids are universally produced by photosynthetic species, but they are also synthesized by heterotrophic organisms, such as many bacteria and fungi [1]. Biosynthetic pathways for carotenoids are less diverse in fungi than in autotrophic species. In most of the cases investigated, it consists of the synthesis of β-carotene or some xanthophylls, such as astaxanthin, torularhodin and neurosporaxanthin [2]. Some fungi, such as *Blakeslea trispora* and *Xanthophyllomyces dendrorhous* (formerly *Phaffia rhodozyma*) are biotechnological sources for the production of β-carotene and astaxanthin, respectively [3], but experimental facilities have made other fungi more suitable organisms to investigate carotenoid metabolism. Two of the best-known fungal models for carotenogenesis, *Neurospora crassa* and *Fusarium fujikuroi*, produce neurosporaxanthin (NX), a peculiar carboxylated carotenoid. This xanthophyll was described in *N. crassa* as an acidic pigment, found in a mixture with other carotenoids, and was later identified as the 35-C carboxylic apocarotenoid β-apo-4′-carotenoic acid [4]. NX provides a typical orange pigmentation to the *N. crassa* cultures, because of its accumulation in the airborne spores, the conidia, and a wealth of information has been generated over several decades on NX production in this fungus [5]. The genetic analyses of albino *N. crassa* mutants led to the identification of the first genes involved in this biosynthetic pathway, opening the investigation of their orthologs in other fungi [2,6].

NX was later also found in *Fusarium* species, such as *F. aquaeductuum* [7], *F. fujikuroi* [8] or *F. oxysporum* [9]. NX is not an abundant carotenoid in nature; in addition to *Neurospora* and *Fusarium*, it has been found in very few fungi, such as *Verticillium* [10,11] and *Podospora* [12]. *F. fujikuroi* is a fungus amenable for biotechnologists [13], and it is well-known as the industrial source of gibberellins, growth-promoting plant hormones. In recent years, *F. fujikuroi* has become also a reference model in the investigation of NX biosynthesis, and much information has been accumulated on the biochemistry and genetics of the pathway [14]. As in *N. crassa*, all the genes and enzymes needed to produce NX have been characterized in *F. fujikuroi*, and clear orthologs are found in the genomes of other *Fusarium* species. The intermediates identified in the carotenoid mixtures in *F. fujikuroi* are consistent with the biosynthetic pathway depicted in Supplementary Material (Figure S1). The first enzyme specifically involved in NX synthesis is phytoene synthase, whose enzymatic activity resides in the carboxy region (A domain) of the bifunctional polypeptide CarRA. Phytoene synthase condensates two geranylgeranyl diphosphate units to produce 15-*cis-*phytoene. This colorless precursor is subject of five desaturations by the enzyme CarB [15], and the cyclization of one end of the molecule by the cyclase domain of CarRA (R domain). The activities of CarRA and CarB lead to the formation of torulene, which is cleaved by the carotenoid oxygenase CarT to produce the apocarotenoid β-apo-4′-carotenal [16], which is finally converted to NX by the aldehyde dehydrogenase CarD [17]. In *F. fujikuroi*, a side branch of the pathway leads to retinal through the activity of a second carotenoid oxygenase, CarX. This enzyme cleaves symmetrically β-carotene, derived from a second cyclization on γ-carotene by CarRA (Figure S1). Retinal is presumably used as chromophore by *F. fujikuroi* rhodopsins CarO [18,19] and OpsA [20,21].

Illumination is a major stimulating factor in *Fusarium* carotenogenesis, and the control of expression of the carotenoid genes by light has received detailed attention [22]. Carotenoid production is much lower without illumination, however mutants overproducing carotenoids are easily identified even in the dark because of their deep orange pigmentation. [9] These mutants, generically called *carS*, have been described in *F. fujikuroi* [23] and *F. oxysporum* [9]. They accumulate large amounts of carotenoids under all tested culture conditions, and contain larger amounts of transcripts for the genes of the carotenoid pathway [14]. Carotenoid production of the *carS* mutants is further enhanced under nitrogen starvation, a regulation that also acts at transcription level [24]. These mutants hold mutations in the gene *carS*, coding for a protein of the RING finger family, and reintroduction of a wild *carS* allele restores their normal carotenoid regulation [9,25]. In addition to being a negative regulator of carotenoid biosynthesis, CarS is involved in the control of a large set of *Fusarium* genes, many of them also regulated by light [26]. The molecular mechanism by which CarS controls carotenogenesis remains to be elucidated, but irrespective of their molecular basis, the *carS* mutants are very useful for the development of carotenoid overproducing conditions.

Indirect evidences suggest a role for carotenoids in the defense against oxidative stress in fungi, both as scavengers of free radicals and quenchers of singlet oxygen [27]. This is probably also the case for NX in *Fusarium* and *Neurospora*, as indicates the induction of NX synthesis in *F. aquaeductuum* and *N. crassa* in the presence of hydrogen peroxide [7,28]. Moreover, mutants of the superoxide dismutase gene *sod-1* of *N. crassa* accumulated more carotenoids than the wild type, following illumination [29]. However, no reports are available on the biochemical and biological properties of NX.

The antioxidant activity of dietary carotenoids or carotenoid-enriched extracts from foods or plants has been extensively evaluated. It has been reported that carotenoids exert a high quenching efficiency against singlet oxygen, [O_2_(^1^Δ_g_)] [30]. Among others, 2,2-diphenyl-1-picrylhydrazyl radical (DPPH) and ferric reducing ability of plasma (FRAP) methodologies are frequently used for measuring in vitro antioxidant activity of biological extracts. The DPPH assay is a radical scavenging methodology based on electron donations of antioxidants to neutralize relatively stable DPPH• radicals, whilst FRAP procedure measures the capability of the antioxidants in the extract for reducing ferric ion (Fe^3+^)-ligand complex to ferrous (Fe^2+^) complex in acidic media [31]. In the last years, a new assay, singlet oxygen absorption capacity (SOAC), was developed to specifically determine the [O_2_(^1^Δ_g_)] quenching capacity of an extract or a pure compound, and the high activity of carotenoids or carotenoids-enriched extracts capturing this reactive oxidizing intermediate was reported [32,33,34,35]. On the other hand, the use of liposomes in antioxidant analysis includes important structural aspects of carotenoids in membranes (localization, organization and orientation) that most of the in vitro antioxidant assays lacks [36]. Within liposomes, it is possible to reproduce some of the carotenoid-phospholipid interactions that occur in natural biological membranes, adding structural features of carotenoids to their inherent scavenging and quenching properties derived from the polyene chains.

This study aims to investigate the antioxidant properties of NX. For this purpose, we have settled suitable overproduction conditions of this xanthophyll by a *carS* mutant of *F. fujikuroi*, and we have studied, by using different in vitro assays and liposomes, the antioxidant activity of cell extracts from the NX-enriched mutant, compared with those from the same strain to which the wild *carS* gene has been introduced, resulting in a very low NX content.

## 2. Materials and Methods

### 2.1. Strains and Culture Conditions

The wild strains IMI58289 and FKMC1995 of *Fusarium fujikuroi* come from the Commonwealth Mycological Institute (Kew, UK) and the Kansas State University Collection (Manhattan, KS, USA), respectively. The *carS* mutants SG22 and SG39 were obtained from IMI58289 by chemical mutagenesis [23] and SF134 from FKMC1995 by the same procedure [17]. SG256 was derived from SG39 by reintroduction of a wild allele of the *carS* gene [26].

To improve carotenoid production, experiments were started in low-N medium, originally 10% ICI medium [37], containing 80 g/L D(+)-glucose, 0.48 g/L NH_4_NO_3_, 5 g/L KH_2_PO_4_, 1 g/L MgSO_4_·7H_2_O, and microelements. Changes in the composition, such as replacement of glucose by sucrose, changes in the nitrogen source, or reduction of KH_2_PO_4_ content are described in the text. The optimized medium used to obtain mycelial samples for antioxidant analysis contained 80 g/L sucrose, 0.5 g/L NH_4_NO_3_, 2.4 g/L KH_2_PO_4_, 1 g/L MgSO_4_·7H_2_O, and microelements from 10% ICI medium.

Mycelia were incubated in 250 mL of culture medium in 500-mL Erlenmeyer flasks. The flasks were inoculated with 10^6^ conidia and grown at 30 °C in the dark on an orbital shaker at 150 rpm. Conidia for inoculation of IMI58289 strains were obtained from mycelia grown for one week on EG agar medium [26] under light at 22 °C. Conidia were collected in water by scrubbing the surface of the cultures with a sterile spatula, filtered through borosilicate filters VitraPOR Filter-Crucible, ROBU*^®^* (Glasfilter-Geräte GmbH, Hattert, Germany) and counted in a haemocytometer (Bürker chamber, Blau Brand, Germany).

To check growth of liquid cultures in flasks, 10-mL samples were filtered through Whatman paper and mycelial biomass was dried and weighed. Samples were also examined under microscope for the presence of conidia. When detected, they were counted in a haemocytometer.

### 2.2. Analyses of Carotenoids

Mycelial samples were frozen at −80 °C and freeze-dried for 24 h. The dry samples were weighed and ground with sea sand in a mortar. Carotenoids were extracted with acetone up to bleaching of the samples, and dissolved in acetone as required, depending on the carotenoid concentration. Neutral and polar carotenoids (NX) were determined by the subtraction protocol, as described [38]. Spectrophotometric measurements were recorded from 350 to 650 nm in a Shimadzu UV spectrophotometer 1800. In the case of the presence of bikaverins, they were precipitated by addition of 1:10 of 10 M NaOH to the acetone extract [39]. The total carotenoid content of SG256 was estimated using the extinction coefficient (E 1%, 1 cm) of the most abundant carotenoid, γ-carotene [7]. Total carotenoid contents in NX-rich samples were estimated using a global extinction coefficient of 2000, except for the analyses carried out in parallel for SG22 and SG39, in which their high NX contents were taken into account to use a global extinction coefficient of 1800. NX concentration in the SG39 and SG256 samples was estimated combining information from the areas of the peaks in the HPLC chromatograms and the spectrophotometric spectra from crude mycelial samples, using a NX extinction coefficient of 1715. Proportions of peak areas in chromatograms displayed in Results for SG39 were 88.7% for peak 1 (NX), 2.4% for peak 2 (torulene), 2.5% for peak 3 (γ-carotene), 1.3% for peak 4 (ζ-carotene) and 5.1% for peak 5 (β-carotene). Percentages in the SG256 chromatogram were 10.6% for peak 1 (NX), 81.8% for peak 3 (γ-carotene) and 7.6% for peak 5 (β-carotene). Accordingly, 88.7% and 10.6% of the crude spectra were attributed to NX in the respective SG39 and SG256 extracts. Following the calculations, NX concentrations were estimated as 8.3 mg/g dry weight in SG39 and 0.003 mg/g dry weight in SG256.

HPLC analyses were achieved as described [40], using a Waters 2695 HPLC equipment, fitted with a reversed-phase C18 column Mediterranea SEA18, 3 µm, 20 × 0.46 cm (Teknokroma, Barcelona, Spain), a Waters 2998 photodiode array detector, and controlled with Empower2 software (Waters Cromatografía, S.A., Barcelona, Spain).

### 2.3. Antioxidant Assays

The antioxidant activity of carotenoid extracts from SG256 (colorless, with trace level of NX) and SG39 (NX-enriched) strains was evaluated in this study by their: (i) reaction with the 2,2-diphenyl-1-picrylhydrazyl radical (DPPH•) (DPPH assay); (ii) ferric-reducing activity in aqueous solution (FRAP assay); (iii) quenching activity against singlet oxygen [O_2_(^1^Δ_g_)] generated in organic solvent (SOAC assay); and (iv) scavenging activity against hydroxyl radicals (HO•) in liposomes.

The chemicals for these assays were obtained from Sigma-Aldrich (Barcelona, Spain), except the fluorescent probe C_11_-BODIPY^581/591^ (Invitrogen, Thermo Scientific, Madrid, Spain), 1,4-naphthalene endoperoxide (EP) (InvitroTech, Kyoto, Japan), β-apo-8′-carotenal (Extrasynthese, Genay, France) and liquid chromatography grade solvents – n-hexane, chloroform, ethyl acetate, methanol, and ethanol – purchased from Merck Co. (Darmstadt, Germany).

Graphics of liposome assays were plotted with software OriginPro 2016, 64-bit, Sr-2.5 (OriginLab Coorporation, Northampton, MA, U.S.A.).

#### 2.3.1. DPPH and FRAP Assays

Freeze-dried mycelia of SG256 (colorless with trace level of NX) and SG39 (NX-enriched) were extracted with 10 mL of 80% methanol or methanol and immediately used for DPPH and FRAP assays, respectively.

For 2-2 diphenyl-1-picrylhydrazyl (DPPH•) determinations, a 100 mM DPPH• methanolic solution was prepared, and 290 µL were mixed with 10 µL of the fungal extract and allowed to stand for 30 min at 27 °C in complete darkness. Absorbance was measured at 515 nm and compared to the absorbance obtained for the control (blank without samples). DPPH• scavenging capacity was expressed as inhibition percentages by the formula:

% DPPH• scavenging capacity = [(^515nm^A_control_ − ^515nm^A_sample_)/ ^515nm^A_control_] × 100

FRAP reagent was prepared by mixing 300 mM acetate buffer, pH 3.6, 10 mM 2,4,6-tripyridyl-*s*-triazine (TPTZ) in 40 mM HCl, and 20 mM FeCl_3_.6H_2_O in a ratio of 10:1:1 (by volume). The methanolic fugal extracts were appropriately diluted in methanol:water 8:1. The FRAP reagent (260 μL) was mixed with 40 μL of the diluted extracts and incubated for 30 min at 37 °C, and then absorbance at 593 nm was read and compared to the absorbance obtained for the control (FRAP reagent, with methanol:water 8:1).

In both DPPH and FRAP assays, the reaction mix was prepared in a 96-well polystyrene microtiter plate (Fisher Scientific, USA) and absorbance measurements were carried out using a SPECTROstar Omega reader (BMG Labtech, Offenburg, Germany).

#### 2.3.2. Quenching Activity

The quenching activity of fungal carotenoid extracts was determined by the Singlet Oxygen Absorption Capacity Assay (SOAC), with some modifications [32,33]. Briefly, freeze-dried mycelial SG256 (colorless with trace level of NX) and SG39 (NX-enriched) were extracted with 5 mL of acetone. Equivalent volume aliquots of SG256 and SG39 acetone extracts were diluted 1:8 in cooled ethanol/chloroform/water mixture (50:50:1, *v/v/v*), and immediately used for assay. In each well of a 96-well quartz microplate equipped with lid (Espectrosil 2000) and PTFE sealing foil (Hellma, Muellheim, Germany) an aliquot of 15 µL was mixed with 150 µL of 0.10 mM 2,5-diphenyl-3,4-benzofuran (DPBF) and 75 µL of 1.5 mM 1,4-naphthalene endoperoxide (EP). DPBF was used as an indicator of the [O_2_(^1^Δ_g_)] quenching capacity, whereas EP generates [O_2_(^1^Δ_g_)] by thermal decomposition in organic solvent. Absorbance changes of DPBF at 413 nm were monitored for 60 min at 35 °C in a microplate SPECTROstar Omega reader. A standard solution of α-tocopherol (αtoc) was used to calculate relative SOAC values as:

[(t_1/2_sample − t_1/2_blank)/(t_1/2_αtoc − t_1/2_ blank αtoc)] × [(αtoc, g/L)/(sample, g/L)]

The estimated concentration of NX in the final reaction mixture of SG39 samples was 21 µM, while in SG256 it was not detectable. For comparative purposes, a standard solution of β-carotene at final concentration of 26 µM in the reaction mixture was evaluated.

#### 2.3.3. Scavenging Activity in Liposomes

Standard solutions of β-carotene (βC 273.3 µg/mL) and β-apo-8′-carotenal (ApoC 283.3 µg/mL), chemical structures included in the scheme depicted in Supplementary Material (Figure S1), were used for comparison with carotenoid extracts from SG256 (colorless) and SG39 (NX-enriched) strains, stored under N_2_ atmosphere at −80 °C and protected from light to avoid oxidation. All solutions and extracts were prepared in chloroform (CHCl_3_). NX concentration in the extracts of SG39 and SG256, determined spectrophotometrically, were 25.2 µg/mL and undetectable, respectively.

For preparation of unilamellar liposomes, egg-yolk phosphatidylcholine (EYPC) was selected for 1.5 mM liposome preparation, since EYPC is a major phospholipid component of biological membranes and contains a significant percentage of polyunsaturated fatty acids (PUFAs), which are the main targets for oxidative damage by free radicals. Standard and fungal carotenoids in CHCl_3_ were previously mixed with EYPC solutions (also in CHCl_3_) to reach a final concentration of 10 µM in the reaction mixture (carotenoid/phospholipid molar ratio <1.0%, to avoid carotenoid aggregation within liposomes). Control liposomes lack carotenoids. Chloroform was subsequently removed from lipid-carotenoid solutions by vacuum in a round-bottom flask adapted to a rotatory evaporator apparatus working at a low speed and moderate heating (≤30 °C), to allow the formation of a homogeneous dried lipid film. The lipid-carotenoid film was stored overnight at −20 °C, in the dark, after flushing N_2_ to avoid oxidation.

In the next day, multilamellar liposomes were prepared by adding 50 mM phosphate saline buffer (PBS), pH 7.5, then agitated in vortex for 5 min, and exposed for 10 min in an ultrasound bath, at room temperature. After lipid-carotenoid film hydration, 25 µL of the free radical-sensitive probe C_11_-BODIPY^581/591^ stock solution, in methanol, were added to multilamellar liposomes, followed by additional 5 min vortex and 10 min ultrasound bath, to reach a final concentration of 10 µM. After that, unilamellar liposomes were prepared by extrusion through 100 nm-pore polycarbonate membranes (MilliPore, Burlington, MA, USA), at 37 °C, in a mini-extruder device (Avanti Lipids. Co., Alabaster, AL, USA). After 15 passes, a clean, transparent and homogeneous unilamellar 1.5 mM EYPC liposomes suspension was obtained and immediately used in the oxidation assays.

Reactive hydroxyl radicals (HO•) were produced in aqueous solution, in the presence of unilamellar liposomes, by mixing 25 mM H_2_O_2_ with 1.5 mM Fe^2+^:6 mM EDTA (Fenton reaction) in 50 mM PBS buffer, pH 7.5, 37 °C. Liposome oxidation was monitored for 170 min by the fluorescence decay at 600 nm of the pre-loaded C_11_-BODIPY^581/591^ probe in liposomes, as aforementioned [41]. Areas above curves (AAC) were integrated from time zero to 170 min (t_0_ and t_170_, respectively) to quantify the extension of membrane oxidation in liposomes.

## 3. Results

### 3.1. Development of Conditions for Improved NX Production

In a previous study, it was found that carotenoid production of *carS* mutants was about 4–5 times higher in a low-N than in a high-N medium [24], indicating that nitrogen is a negative regulator of the pathway. Since the *carS* mutants accumulate carotenoids constantly during cultivation [23], incubations of up to five weeks were carried out in low-N medium using the *carS* mutant SG22, obtained from the wild strain IMI58289 [23]. Previous experiments of the research group have used another wild strain, FKMC1995, which exhibits a better sporulation than IMI58289 on solid medium. The *carS* mutant SF134, previously obtained from this strain [17], was cultured in parallel with SG22. Both strains hold mutations in conserved residues of the *carS* gene [25].

**Figure 1:**
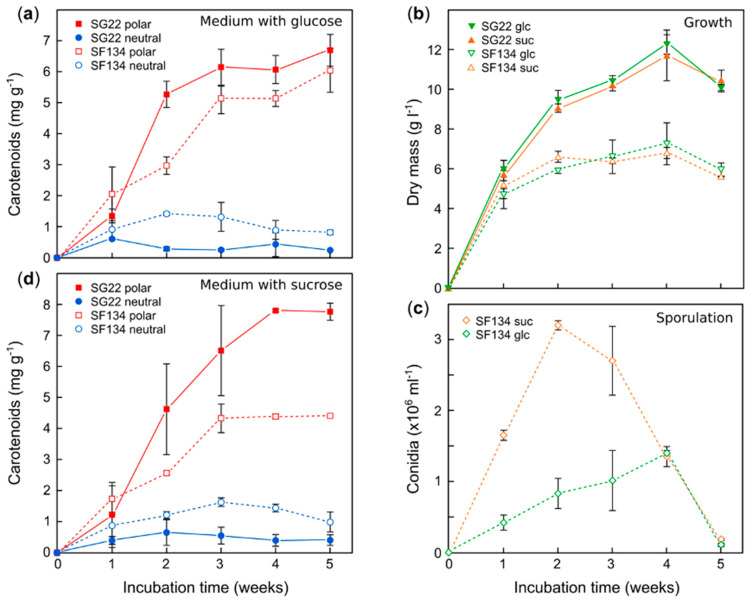
Effect of carbon source on carotenoid production, growth and conidiation in low-N medium. Time course of carotenoid production (**a**,**d**), growth (**b**), and conidia concentration (**c**) in flask cultures of the *carS* mutants SG22 and SF134 in low-N medium with glucose or with the same amount of sucrose as carbon source. The data show the average and standard deviations from two determinations.

The results showed an increase in carotenoid content during the first three weeks of culture (Figure 1a), reaching approximately 6 mg of carotenoids/g dry mass. The biomass kept growing, however, for 4 weeks (Figure 1b). Carotenoid production was lower in the mutant SF134, which also exhibited less biomass in the cultures (Figure 1b). This last result may be due to the dedication of resources to the production of conidia, which were detected in the SF134 cultures, but not in those of SG22 (Figure 1c). Most carotenoids accumulated in these culture conditions were polar, basically NX, as indicated the absorption spectrum of the samples. Not only the amount of carotenoid, but also the proportion of NX were higher in SG22 than in SF134.

The medium used for this experiment contains glucose as a carbon source. Previous results showed that sucrose is a more efficient carbon source for the synthesis of other metabolites, such as bikaverin [42]. Therefore, experiments were performed in parallel with the two strains using sucrose instead of glucose. The results were similar to those obtained in glucose, except that the production of the SG22 strain was more efficient with sucrose (Figure 1d). Therefore, sucrose was used hereafter as a carbon source.

**Figure 2:**
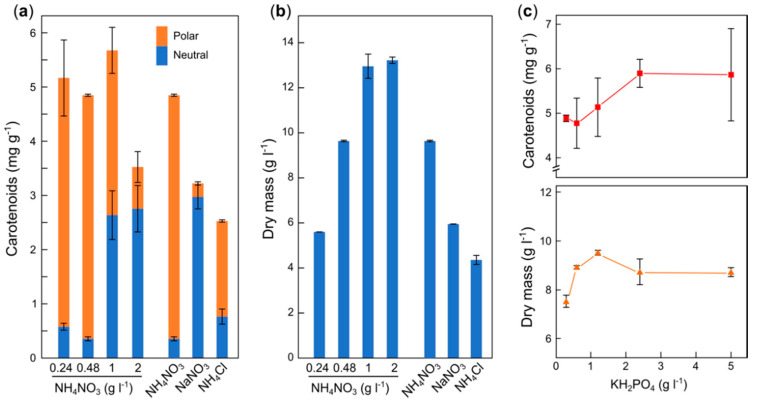
Carotenoid production and growth in the mutant SG22 under different culture conditions. (**a**,**b**) Effect of nitrogen on concentrations of neutral (blue lower bars) and polar (orange upper bars) carotenoids and mycelial mass in the cultures. The strain was cultured in low-N medium with 80 g/L sucrose as carbon source and with the indicated nitrogen sources. The three bars on the right correspond to 0.48 g/L of NH_4_NO_3_, 1 g/L of NaNO_3_, and 0.64 g/L of ClNH_4_, respectively. (**c**) Effect of phosphate on carotenoid content and growth. Low-N medium contained sucrose instead of glucose and the indicated KH_2_PO_4_ concentration. The data show the average and standard deviations from two determinations.

The nitrogen source of the low-N medium was NH_4_NO_3_, and its concentration, 0.48 g/L, was low enough to be the limiting element, and therefore the first to run out during cultivation. Further experiments tested with different concentrations of NH_4_NO_3_ showed that the production of carotenoids decreased when exceeding 1 g/L of NH_4_NO_3_ (Figure 2a). Below 2 g/L, the biomass was proportionally lower, as less nitrogen was used (Figure 2b), as expected to be the limiting factor for growth. Although the highest amounts of total carotenoids were obtained with 1 g/L, the proportion of NX was lower, presumably due to lower aeration. Taking mycelial growth and NX production into account, we chose 0.48 g/L as an adequate NH_4_NO_3_ concentration. Since NH_4_NO_3_ provides two atoms of nitrogen per molecule, NaNO_3_ and NH_4_Cl were also tested in equimolar amounts of N at 0.48 g/L of NH_4_NO_3_. Lower growth and carotenoid yields were observed with these nitrogen sources, and even a much lower proportion of NX in the case of NaNO_3_.

The culture medium contains a high concentration of phosphate (5 g/L of KH_2_PO_4_). Information on phosphate dependence for carotenoid production in *Fusarium* is missing, and therefore we tested lower concentrations of this compound (Figure 2c). To facilitate calculations, we decided to use hereafter 0.5 g/L NH_4_NO_3_ as nitrogen source. The results showed growth inhibition only at phosphate concentrations below 0.5 g/L, indicating that phosphate is in excess in culture medium. Nevertheless, the concentration of carotenoids dropped below 2.4 g/L of phosphate, and therefore this was considered a suitable phosphate concentration for carotenoid production purposes.

### 3.2. Obtaining of NX-Enriched Extracts for Antioxidant Assays

**Figure 3:**
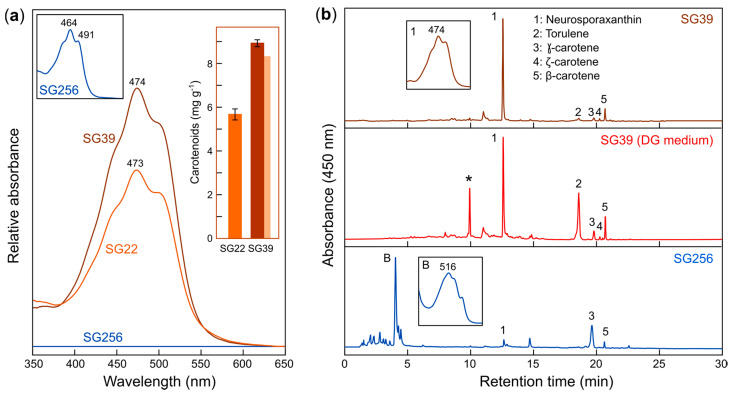
Analysis of carotenoids in the SG22, SG39 and SG256 mycelial samples. (**a**) Absorption spectra of the cell extracts of the indicated strains. No absorption was detected for SG256 at the scale used for SG39 and SG22. The spectrum of a concentrated SG256 sample is shown in the left inner graph. Wavelength of spectrum peaks are indicated (nm). Carotenoid concentrations in SG22 and SG39 mycelia are represented in the right inner graph. Estimated neurosporaxanthin (NX) content in SG39 is indicated by the paler band shown behind. (**b**) Chromatogram of the HPLC separation of the carotenoids accumulated by the SG39 and SG256 strains of *F. fujikuroi* (detection wavelength at 450 nm). UV/Visible spectrum of the major peak is shown in the inner box. Relative areas of labeled peaks for SG39 and SG256 are indicated in Materials and Methods. B: bikaverin peak and spectrum. For comparison, the chromatogram of a sample from SG39 grown under standard culture conditions (DG minimal medium) is also shown [14]. The asterisk corresponds to an unknown carotenoid.

To check the antioxidant properties of NX, we took advantage of the high NX content of extracts of *carS* mutants of *F. fujikuroi* grown in Low-N medium with 80 g/L sucrose, 0.5 g/LNH_4_NO_3_ and 0.5 g/L of KH_2_PO_4_ (optimized medium). The available *carS* mutants were obtained by mutagenesis, and they may carry other secondary mutations. In a recent study about the effect of the *carS* mutation on the *Fusarium* transcriptome, a different *carS* mutant, SG39, was used in parallel with a complemented SG39 with the wild allele, called SG256, with low carotenoid content [26]. To test if SG39 was equally efficient under our conditions for high NX production, SG22 and SG39 and SG256 were grown in optimized medium. The results indicated that SG39 had even higher carotenoid production capacity than SG22, with similar crude spectra, but with carotenoid levels reaching about 9 mg/g dry mass (Figure 3a). In contrast, as expected from the complementation of the *carS* mutation in SG39, SG256 cultures contained very low amounts of carotenoids, ca. 0.018 mg/g dry mass, that is, ca. 400-fold less than the original SG39 mutant. Moreover, the absorption spectrum of SG256 biomass suggested the presence of minor amounts of yellowish carotenoids, with shorter absorption maxima than NX (inner spectrum in Figure 3a).

HPLC analysis of the carotenoids accumulated by SG39 confirmed that NX is the major carotenoid under these culture conditions, whereas the NX precursors were found at very low concentrations (Figure 3b, upper chromatogram). The combination of spectrophotometric and HPLC data allowed us to estimate that about 89% of carotenoid absorption corresponds to NX (details in Materials and Methods), resulting in about 8.3 mg NX/g dry mass. Among the minor carotenoids, β-carotene predominated in SG39 extracts. This carotenoid pattern contrasts with previous analysis from the same strain growing on DG minimal medium, routinely used in our laboratory as standard culture conditions, which contains higher amounts of NX precursors (Figure 3b, second chromatogram). As expected from its crude spectrum, the *carS*-complemented strain SG256, contains only traces of carotenoids (Figure 3b, lower chromatogram), with γ-carotene as the major carotenoid and a very low NX content, estimated as 0.003 mg/g dry mass. A peak at a low retention time in the SG256 chromatogram, labeled as B, has an absorption spectrum (inner box) consistent with bikaverin [42]. This polyketide pigment is expected to be also present in SG39, but its occurrence is masked by the 400-fold higher concentration of carotenoids in this strain. As a result of the drastic differences in their NX content, extracts from SG39 and SG256 cultures obtained under these conditions were further used for antioxidant assays.

### 3.3. Antioxidant Assays

**Figure 4:**
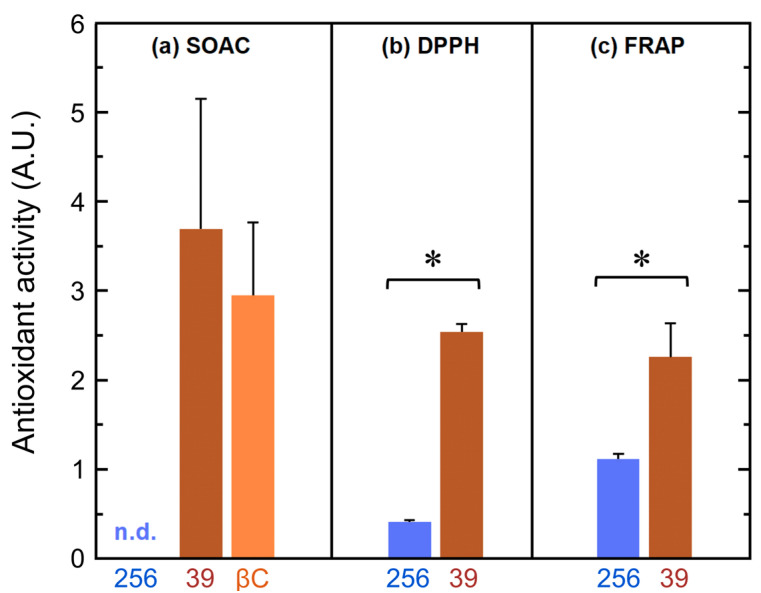
Antioxidant activities of extracts from SG39 (NX-enriched) and SG256 (NX-poor control) fungal strains against: (**a**) singlet oxygen [O_2_(^1^Δ_g_)], by quenching activity in singlet oxygen absorption capacity (SOAC) assay; (**b**) 2,2-diphenyl-1-picrylhydrazyl radical (DPPH•) radical in aqueous solution; and (**c**) Fe^2+^-mediated free radicals in aqueous solution, by ferric reducing ability of plasma (FRAP) assay. Abbreviations: 256, SG256; 39, SG39; βC, β-carotene; n.d.: not detectable; A.U.: arbitrary units. Data are presented as mean and ± standard deviation, (*x* ± SD, *n* ≥ 3). * *p* < 0.05 (statistical analysis performed with the *t*-Student’s test at significance level of 5%).

**Figure 5:**
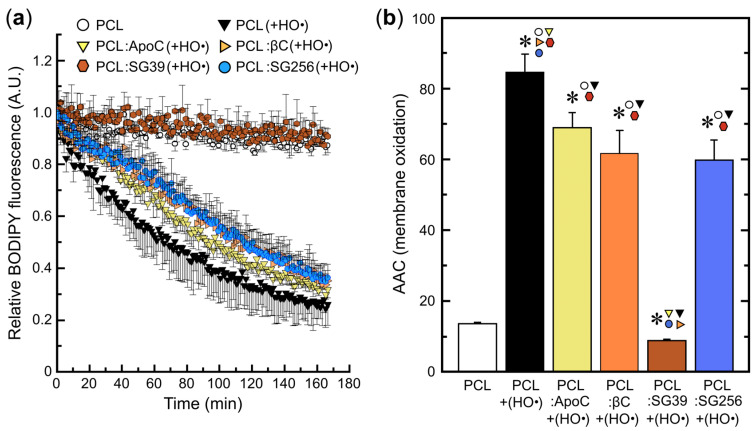
Membrane oxidation in liposomes. (**a**) Fluorescence kinetics of C_11_-BODIPY^581/591^ in 1.5 mM egg-yolk phosphatidylcholine liposomes (PCL) with 10 µM β-carotene (PCL: βC), β-apo-8′-carotenal (PCL:ApoC), NX-rich SG39 extracts (PCL:SG39), and NX-poor SG256 extracts treated with 25 mM H_2_O_2_ and 1.5 mM Fe^2+^:6 mM EDTA, in 50 mM phosphate-saline buffer (PBS), pH 7.5, 37 ^o^C; (**b**) areas above curve (AAC) of fluorescence decay kinetics—as an index of liposome oxidation—from t_0_ to t_170_ min presented in Figure 5A. Statistical differences of the bars against those indicated by the colors of the symbols are shown above (* *p* < 0.05).

The antioxidant activity of SG39 and SG256 extracts was initially determined by measuring their quenching activity against [O_2_(^1^Δ_g_)] (SOAC assay, Figure 4a). Later, the scavenging activities of fungal extracts were measured by DPPH• and FRAP methodologies in methanolic:aqueous solutions (Figure 4b,c, respectively), and by scavenging HO• radicals in liposomes (Figure 5). Altogether, SG39 extracts showed significant higher antioxidant activity than SG256 extracts: 6-fold higher DPPH• scavenging activity, 2-fold higher FRAP capacity and comparable SOAC scores with β-carotene, whereas no SOAC activity was detected in SG256 extracts.

Control experiments were carried out to characterize the oxidation pattern of C_11_-BODIPY^581/591^ in EYPC liposomes under 25 mM H_2_O_2_ + 1.5 mM Fe^2+^ treatment (Supplementary Material, Figure S2). As shown in SM2, the liposomes are promptly oxidized by Fe^2+^ addition, although a slight spontaneous oxidation is also evident in the presence or absence of 25 mM H_2_O_2_, both in the absence of the redox catalyst Fe^2+^. As additional controls, no differences were observed between any carotenoid:liposome system and EYPC liposomes (without carotenoids or fungal extracts) in the absence of Fe^2+^ to trigger the Fenton reaction (data not shown).

The index of membrane oxidation was determined by the fluorescence decay of C_11_-BODIPY^581/591^ probe (Figure 5a) in 1.5 mM EYPC liposomes containing either 10 µM β-carotene (PCL: βC), 10 µM β-apo-8′-carotenal (PCL:ApoC), or 10 µM NX-enriched SG39 (compared to SG256) extracts by HO• radicals formed from the Fenton reaction (25 mM H_2_O_2_ + 1.5 mM Fe^2+^:6 mM EDTA) in 50 mM PBS buffer, pH 7.5. The extension of membrane oxidation was quantified from t_0_ to t_170_, as the area above curves of Figure 5a in all liposomal systems (Figure 5b). NX-rich SG39 extracts prevented liposome oxidation triggered by HO• radicals in aqueous solution, whereas PCL:βC (−27%), PCL:ApoC (−19%), and SG256 extracts (−30%) partially inhibited the progression of membrane oxidation, as depicted in Figure 5b.

## 4. Discussion

Carotenoids are pigments with a high biotechnological potential, because of their applications as healthy food supplements. Some carotenoids have specific beneficial effects because of their functions as source of vitamin A or as eye macular pigments. Many other carotenoids do not comply with these functions, but all of them are assumed to be beneficial, because of their antioxidant effects [43], which vary depending on their specific chemical features. The antioxidant properties of food carotenoids have been widely investigated, but many others await to be investigated. Thus far, no research has been carried out on the antioxidant effects of NX, a carboxylic xanthophyll only found in some fungi. Notably, some previous experimental indications point to antioxidant roles of NX in producing fungi. However, NX is not found in very large amounts in wild strains of such fungi. Thus, about 0.1–0.2 mg/g of a NX-containing carotenoid mixture are found in mycelia grown under light in *F. fujikuroi* [23], *N. crassa* [44] and *P. anserina* [45]. In the case of *N. crassa*, carotenoids are accumulated in higher amounts in their conidia [46], providing a typical intense orange pigmentation to their aerial cultures.

Carotenoid overproduction by microorganisms is an advantageous feature for biotechnologists. In some cases, this is attained using appropriate culture conditions, such as intense solar exposure and other stress conditions for β-carotene or astaxanthin-producing microalgae [47]. Improved production conditions are also facilitated using carotenoid overproducing strains. NX overproducing mutants, typically producing about ten-fold the amount of the wild strain, are well known in *F. fujikuroi* [23,25] and *F. oxysporum* [9]. Irrespective of the molecular basis of this deregulation, still under investigation, these mutants constitute a valuable tool for biotechnologists to develop industrial conditions to obtain significant amounts of this unusual carotenoid. NX overproducing mutants have been also described in *N. crassa*, such as *ovc* and *vvd*, but their carotenoid levels are not so high as those of *Fusarium carS* strains [48]. Former data indicated an activating effect of nitrogen starvation on NX biosynthesis in *N. crassa* [49] and *F. fujikuroi* [24]. In this work we have shown that the culture of a *carS* mutant under nitrogen limiting conditions allow to reach NX levels as high as 8 mg/g dry mass. This is the highest NX concentration so far described in a fungus, and by extension in any biological system. Moreover, the carotenoid-enriched mycelial samples obtained under these conditions have an unusually low proportion of NX precursors, facilitating their use to study the properties of this xanthophyll. Carotenoid overproduction has been attained also in other NX producing fungi by overexpression of key genes of the pathway, such as the early HMG-CoA reductase gene in *N. crassa* [50] or the phytoene synthase/cyclase and desaturase genes in *P. anserina* [12]. However, the carotenoid contents described in these works were well below the levels reached in our cultures and have lower NX proportions. However, similar strategies could allow greater increases in NX content in *carS F. fujikuroi* mutants, and some of them are currently being investigated.

Here, we have used NX-enriched *F. fujikuroi* samples (SG39) obtained under our high-producing conditions, in comparison to parallel NX-poor samples obtained from a strain with the same genetic background (SG256), to evaluate NX antioxidant activity. Altogether, data reveal that SG39 extracts have substantially higher antioxidant capacity than SG256 extracts, both considering their quenching activity against [O_2_(^1^Δ_g_)] in organic solvent, and their scavenging activity against different free radicals in aqueous solution and in liposomes (Figure 4 and Figure 5). Since carotenoids were essentially absent in SG256 strain (Figure 3b), the baseline antioxidant activity detected in SG256 may be attributed to other non-carotenoid components in the fungus biomass, such as flavonoids, α,γ-tocopherols, ascorbic acid, and even exopolysaccharides [51]. Although SG39 strain also contains those antioxidants, the substantial accumulation of NX in SG39 (with minor contribution of other carotenoids) may account for its remarkable antioxidant capacity compared to SG256.

The observed quenching activity of SG39 extracts in organic solvent (SOAC assay) is a result of the inherent capacity of NX and other minor carotenoids (including molecular interactions between them) to suppress excitatory energy from [O_2_(^1^Δ_g_)]. Therefore, the observed quenching activity here is directly related to their peculiar electronic-conjugated polyene structure, as all carotenoids are loosely diffusing in organic solvent, according to SOAC methodology [33]. For comparative purposes, the relative SOAC value for a standard solution of β-carotene was also determined (Figure 4a), and it was 29% lower than SG39 scores; however, the differences in the relative SOAC values between SG39 and β-carotene were not statistically significant. It cannot be confirmed that NX simply has comparable quenching capacity than β-carotene, since it cannot be afforded the contribution (even synergism) from other carotenoids present in the samples, or even the interactions between them and NX in SG39 extracts [52]. It has been described that carotenoids are particularly highly efficient quenching [O_2_(^1^Δ_g_)] [30], about two to three orders of magnitude higher than tocopherols [53], catechins and other phenols [54]. Therefore, it was not surprising that SG256 extract, containing only traceable amounts of carotenoids, showed no significant quenching activity compared to SG39 in SOAC assays (Figure 4a). The quenching activity of SG39 extracts correlates well with those found in fruits and vegetable products, and with their regular carotenoid content [55]. There are no available data for the SOAC values of NX, only for other dietary carotenoids such as lycopene, astaxanthin and β-carotene, with lycopene being the carotenoid with the highest relative SOAC values observed [35]. Based on the relative SOAC values obtained here, the quenching capacity of NX could be compared to that of lycopene (or even higher), which is considered as the most efficient [O_2_(^1^Δ_g_)] quencher in biological systems [56].

Apart of carotenoid activity in organic solvent, the DPPH• and FRAP assays addressed the capacity of carotenoids to trap different radicals in polar media (methanolic and aqueous solutions, respectively). These properties, again, depend on the conjugated polyene structures of carotenoids (and attached groups), but also on the reactivity and diffusion of those radicals in the reaction milieu [57]. Regarding the hydrophobicity of the extended polyene chain of carotenoids, the possibility of the molecular aggregation of carotenoids under the experimental conditions described by the FRAP and DPPH• methods cannot be excluded, and this would mask at least part of their actual scavenging properties (specifically SG39, in this case) [58,59]. Interestingly, the antioxidant activity of NX-rich SG39 extracts obtained by the DPPH• assay (in the less polar solvent methanol), was substantially higher than those obtained by the FRAP method in aqueous solution (Figure 4b,c). Moreover, the FRAP assay includes the iron-chelating capacity of tested compounds, which, therefore, also reveals their activity as ‘preventive’ antioxidants by limiting the metal-catalyzed formation of free radicals in aqueous solution [60]. Provided with a carboxyl group (Figure S1), NX would stand as a potential chelating molecule for Fe^2+^ ions (and possibly Fe^3+^, as well), affecting free radical formation in aqueous solution [61]. Accordingly, astaxanthin, a keto-hydroxy xanthophyll, presents red shifts in its maximum absorbance bands upon complexation with Cu^2+^, Cd^2+^, Zn^2+^, Ca^2+^, Pb^2+^, and Hg^2+^ ions, which reflects changes on astaxanthin electronic arrangement and, putatively, on its antioxidant properties [62]. Inter- and intramolecular hydrogen bonds, as well as the medium pH, are also very relevant factors to determine the antioxidant/scavenging properties of carotenoids, and especially xanthophylls in aqueous systems [63,64].

The scavenging activity of SG39 extracts was tested against HO• radicals in liposomal systems. In liposomes, it is presumed that NX and other carotenoids in fungi extracts will assume a proper spatial orientation across the lipid bilayer and, thus, mimic the carotenoid-phospholipid interactions that occur in biological membranes. Therefore, the localization, organization and orientation of carotenoids within lipid bilayers (structural issues) will be accomplished in the liposomal system, in addition to their inherent scavenging properties derived from the polyene chain. It is worth noting that the present study used low carotenoid concentrations in liposomes, limiting it to <1% mol carotenoid/lipid, in order to assure monomeric organization of carotenoid molecules embedded in the biomembranes [65]. Figure 5 depicts the substantial inhibition of liposome oxidation promoted by NX (and other minor carotenoids) in SG39 extracts. In fact, it could not be observed any oxidation of the fluorescence probe C_11_-BODIPY^581/591^ in EYPC liposomes under these circumstances. Interestingly, standard carotenoids βC and ApoC, and SG256 extracts only showed partial inhibition of liposome oxidation (−27%, −19% and −30%, respectively), but we should be careful when comparing these effects, since carotenoid incorporation yields were not calculated in our study [66]. However, lower carotenoid:phospholipid ratios, as those used in our experimental conditions (<1% mol), avoid aggregate formation and assure more proximal incorporation yields of carotenoids into liposomes [66]. Once again, it is plausible that the carboxyl group on NX molecule could chelate Fe^2+^ ions and hamper their participation as catalysts of the Fenton reaction in aqueous solution. However, taking NX (ca. 10 µM) and Fe^2+^ (1.5 mM) concentrations into account, most of the scavenging effect observed in SG39 should be related to its peculiar electronic structure (Figure S1) and related reactivity against HO•. As free radical scavengers, carotenoids react with reactive oxygen/nitrogen species (ROS/RNS) by three distinct mechanisms: (i) radical addition/adduct formation, (ii) electron transfer, and (iii) allylic hydrogen abstraction [67]. All mechanisms involve the formation of carotenyl radicals (Car•) and further peroxy-carotenyl radicals (CarOO•), the stability of which is directly associated with the extension of the electronic conjugated polyene chain (by resonance). Interestingly, Krasnokutskaya et al. [68] demonstrated that insertion in the polyene molecule of a carbonyl group results in a drastic decrease in the reactivity towards molecular oxygen (autooxidation), and, consequently, the formation of CarOO• and Car• radicals (this, during the propagation step of lipid oxidation chain reaction). The authors showed that the reactivity of carboxylated carotenoids decreases, due to the lower initiation and propagation rates in the free radical chain reaction [68]. Notably, ApoC (β-apo-8′-carotenal), a shorter apocarotenoid (C30) with an aldehyde group (Supplementary Material, Figure S1), does not account for such an extended conjugation system as the carboxylated NX, and merely showed equivalent scavenging properties as βC (lacking any conjugated polar group) in liposomal systems here (Figure 5A). Further experiments are necessary to fully understand the impact of carboxylic acid insertion, versus other carbonyl and polar groups, in carotenoids and their scavenging properties in lipid bilayers.

## 5. Conclusions

NX is an unusual C35 carboxylic xanthophyll found only in some filamentous fungi. Since it is absent in human diet, its antioxidant properties have received no attention. For this purpose, it is convenient to have a biotechnologically appropriate NX source. Here, we show that carotenoid overproducing mutants available in the fungus *F. fujikuroi*, affected in the negative regulator CarS, may be a suitable biotechnological source to obtain this carboxylic carotenoid. Using a medium with high sucrose concentration and a limiting amount of nitrogen, we found culture conditions that allow the production of *F. fujikuroi* biomass with a concentration of NX reaching 8 mg/g dry mass, with only minor amounts of NX precursors. These samples were used for in vitro assays, to check the antioxidant properties of NX in comparison with parallel fungal samples with an extremely low NX content. The results showed significant quenching activity of NX-rich extracts against [O_2_(^1^Δ_g_)] in organic solvent assays, (SOAC), which were at least as efficient as β-carotene. Similarly, the NX-rich extracts showed high scavenging activity against HO• radicals in liposomal systems. Although more tests are needed to confirm NX efficacy, in overall, these data are very promising regarding a future use of NX as a carotenoid with beneficial properties as a feed or food additive.

## Supplementary Materials

The following are available online at https://www.mdpi.com/2076-3921/9/6/528/s1, Figure S1: Carotenogenesis pathway in *F. fujikuroi*; Figure S2: Fluorescence kinetics of C_11_-BODIPY581/591 in 1.5 mM treated EYPC liposomes (PCL).
