# Supplementary material for: Neurosporaxanthin Overproduction by Fusarium fujikuroi and Evaluation of Its Antioxidant Properties
Source: Antioxidants (Basel). 2020 Jun 16;9(6):528. doi: 10.3390/antiox9060528 (PMC7346100; doi:10.3390/antiox9060528)
Supplement: Supplementary file 1 [file antioxidants-09-00528-s001.pdf]

antioxidants

Neurosporaxanthin overproduction by *Fusarium fujikuroi* and evaluation of its antioxidant properties

Obdulia Parra-Rivero, Marcelo Paes de Barros, María del Mar Prado, José-Vicente Gil, Dámaso Hornero-Méndez, Lorenzo Zacarías, María J. Rodrigo, M. Carmen Limón, and Javier Avalos

Supplementary Materials

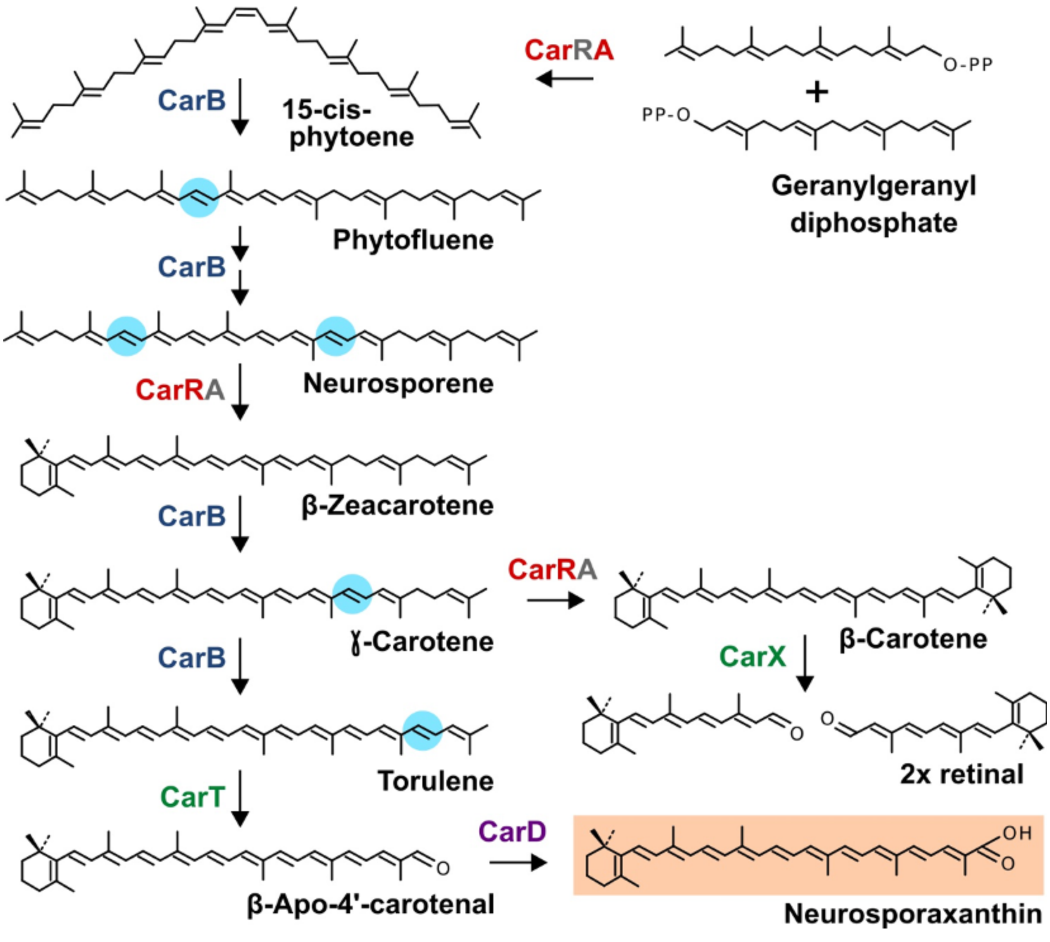

Figure S1. Carotenogenesis pathway in *Fusarium fujikuroi*. Enzymes responsible of each step are indicated according to their gene denominations. Desaturations are indicated in blue. The major product, neurosporaxanthin (NX), is highlighted in orange.

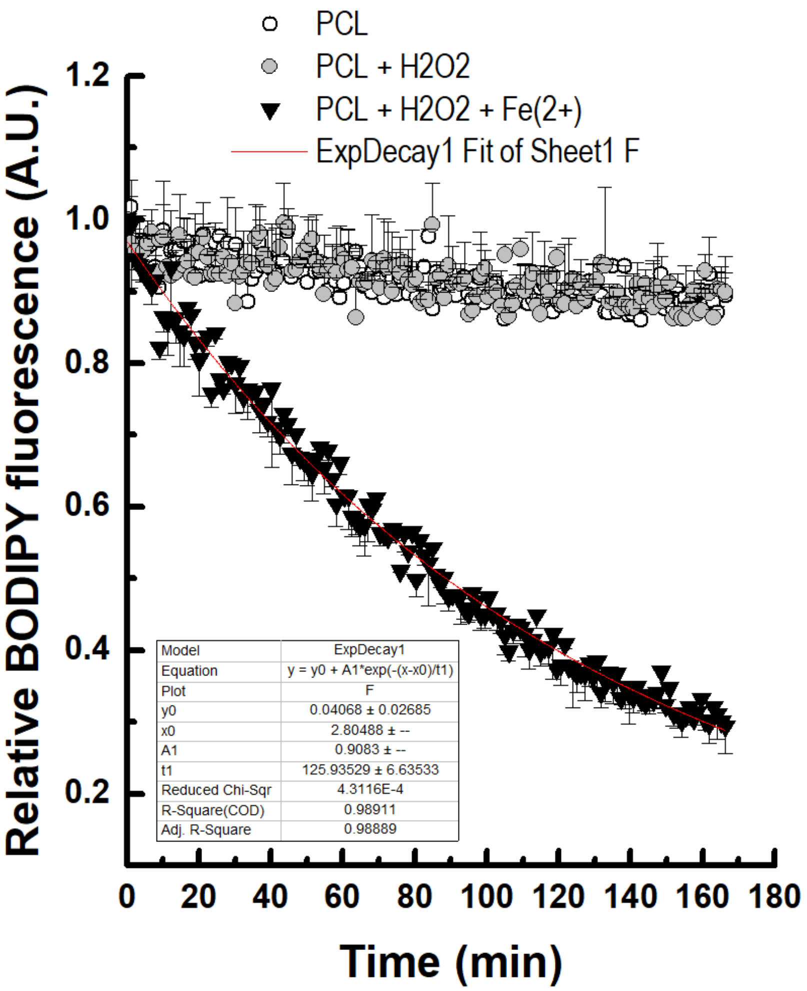

Figure S2. Fluorescence kinetics of C11-BODIPY581/591 in 1.5 mM treated EYPC liposomes (PCL). Treatment: 25 mM H_2O_2 and 1.5 mM Fe^{2+}:6 mM EDTA, in 50 mM phosphate-saline buffer (PBS), pH 7.5, 37°C.
